# Observations upon Those Appearances Which Have Been Supposed to Arise from the Action of the Gastric Juice upon the Stomach Post Mortem. With Cases in Illustration, Taken from the "Allgemeine Medezinische Annalen," Viertes Heft

**Published:** 1824-12

**Authors:** John North

**Affiliations:** Surgeon.


Art. II.—*Observations upon those Appearances which have been supposed to arise from the Action of the Gastric Juice upon the Stomach post Mortem. With Cases in illustration, taken from the "Allgemeine Medezinische Annalen," viertes heft.* By John North, Surgeon.

Some interesting discussions have lately taken place, in the most respectable medical Societies of the metropolis, upon the occasional detection of a partial or total destruction of the stomach, and contiguous parts, in young children. I am not aware that any cases have been published in this country, in which a satisfactory detail of the symptoms, which were in such instances exhibited during life, has been given.‡ I have therefore translated the three following cases from a German Journal: to me they appear to possess much interest. If it should be objected that the symptoms are detailed with unnecessary prolixity, let it be remembered that what is termed German minuteness bears also the stamp of German accuracy and industry.

Case I.—A feeble boy, eighteen weeks old, who had been lately weaned, was attacked with the thrush. The infant was thirsty, emitted an unpleasant sour smell from the mouth, vomited frequently, and had seven or eight stools a-day. The matter that was passed by vomiting and by stool, was of a thin slimy nature. The little patient screamed frequently, and towards evening the skin became hot. The disease was considered to be an inflammatory irritation in the mucous surface of the stomach and bowels, produced by indigestible food. The health

‡ At the time of my writing this paper, I did not know that a very luminous account, containing an abstract of many cases of a similar kind, had been published by Dr. Gairdner, in the Transactions of the Medico-Chirurgical Society of Edinburgh, 1824. Dr. Gairdner supports the Hunterian doctrine, of the destruction of the stomach being caused by the action of the gastric juice. His paper is highly interesting, and deserves an attentive perusal.

of the child was soon apparently restored, by giving half a grain of calomel every three hours, with a little sulphate of magnesia. Borax was applied to the thrushy parts; and the diet consisted of weak broths and milk, with fennel tea. After a few weeks of good health, the child was found, on awaking from a tranquil sleep, to be affected with a rattling in the throat: the limbs were convulsed; the face was suddenly flushed; and, before any physician could be procured, he had expired.

Upon opening the body, the following appearances were observed: —Upon sawing through the bones of the cranium, a considerable quantity of clear water poured forth. The brain was soft and pulpy; its vessels rather turgid *(blutreich)*; the ventricles also contained more than the natural quantity of water. The stomach, in several places, presented a reddish brown and pulpy appearance: there were no apertures in any of the softened parts, but the slightest handling of them made holes, through which was discharged a sour-smelling fluid, which had been abundantly contained in the stomach.

CASE II.—A boy of a year old, weaned but a short time, had only lately recovered from cough, which was consequent to measles. For six weeks he had been affected with diarrhœa, passing six or eight stools a-day: the evacuations consisted of a thin, yellowish green, and lumpy matter. The infant was hot, thirsty, and slept unsoundly; the legs were frequently drawn towards the belly, with screaming. These symptoms increased in severity; the diarrhœa became more frequent; the child was pale, emaciated, drank greedily, screamed frequently, and the skin was parched. Violent vomiting soon occurred. Professional assistance was now first sought. The little creature had a blanched appearance; the eyes were sunken and dull; it groaned and whimpered lowly, lay almost motionless upon the back, breathed deeply and slowly, but occasionally roused from this state of apathy with a scream; threw itself about in a restless manner; drew up the feet towards the belly, struck the abdomen frequently with the hands, which were then with violence directed to the nose and mouth; and, after this state of excitement, the previous appearance of tranquillity was resumed. The vomiting still continued frequent; the head, belly, and palms of the hands, were hot to the touch; no perspiration was observed; the tongue was white and moist; a sour smell proceeded from the mouth; the thirst appeared almost insatiable. As often as the child drank a little milk, it threw up a sour smelling, thin, slimy fluid, with lumps of the milk coagulated. Pressure upon the belly appeared to give no pain, but it caused slight vomiting. In the evacuations from the bowels, which were frequent, a thin watery fluid, mingled with small, yellow lumps of fæces, was discharged. Urine was seldom passed. The pulse was small and irregular, and beat 120 in a minute. The child died in the night, after such means had been recommended as appeared appropriate to the above state.

Upon examining the body, the venous system of the brain was found loaded with blood. The lungs here and there strongly adhered to the pleura, probably from the effect of measles. The stomach and all the small intestines were in the highest degree of a pulpy softness: the cardiac extremity of the stomach was perforated, and through this preternatural opening a very considerable quantity of a sour, yellow, thin fluid, mingled with lumps of coagulated milk, had escaped into the cavity of the abdomen. Much more of the same fluid was contained also in the stomach and small intestines. So thin were the small intestines, that the slightest touch made a hole in them: they presented, as did also the stomach in different places, an appearance of a darkish colour. Well-defined traces of inflammation were not perceived.

CASE III.—A boy, three years and a quarter old, who had been weaned at too early an age, and over-fed with indigestible food, became restless, and frequently vomited a thin, slimy, and very sour-smelling fluid; and also passed each day as many as from eight to ten thin, slimy stools, of a very offensive smell. Whatever was taken into the stomach was succeeded by vomiting. Great thirst was present; the tongue was white; the breath had an acid smell; the belly was considerably swoln, and appeared painful in the epigastric region. There was an occasional discharge of very fetid flatus. The child cried frequently, and often carried the hands towards the mouth. There was slight fever, with occasional sweats. The means employed—sal-ammoniac *(salmiak)*, gum arabic, a little extract of poppies, starch glysters, and warm fomentations, were not productive of any benefit. The vomiting, diarrhœa, the tenderness of the belly, the restlessness, the constant crying and moaning, increased; and, on the sixth day of the disease, the fatal event occurred.

Upon opening the head, the bones of the cranium were perceived of a bluish-red colour, from the turgescence of the veins. The vessels of the dura mater were over-loaded with blood, and the whole texture of the brain was highly vascular. The lateral ventricles contained air, which had somewhat raised up their covering; they contained also a small quantity of reddish serum; and still more of this fluid was found in the basis of the skull, and in the vertebral canal. The cardiac extremity of the stomach had a brownish-yellow and gelatinous appearance, and presented here and there a black colour: in it there was a considerable aperture, through which a yellowish and remarkably sour-smelling fluid had passed into the cavity of the abdomen. Some of the same fluid also remained in the stomach; the whole texture of which was so extremely tender, that it ruptured upon the slightest touch.

To the German and French physicians, cases similar to the above present, I believe, but little novelty. In children particularly, destruction of the stomach and bowels has frequently been detected by them. It is probable that the frequent occurrence of the appearances above described may have escaped the observation of English practitioners, from their want of equal anatomical zeal. The symptoms by which these cases were characterised, would not excite a suspicion of any other affection than a mere derangement of stomach and bowels, and an inspection of the body would not be considered necessary. I have myself seen one example of a similar kind, which I published some years ago.* The stomach is in the museum of Mr.

* *Medical Repository*, August 1815.

Brookes. The symptoms under which the child laboured, were nearly similar to those above described. It cannot be doubted that these appearances in the stomach and intestines precisely resemble those which were supposed by Mr. Hunter to arise from digestion of the stomach after death. Mr. Hunter describes the appearances he observed, in these words:—" The edges of the opening appear to be half dissolved, very much like that kind of dissolution which fleshy parts undergo when half digested in a living stomach, or when dissolved by a caustic alkali,—viz. pulpy, tender, and ragged." The explanation of Mr. Hunter has to me always appeared very unsatisfactory; the cases above related confirm me in the doubts I previously entertained. In the paper to which I have alluded, I have stated some reasons, which seem to me to render Mr. Hunter's supposition extremely improbable. Mr. Hunter* believed that what he termed digestion of the stomach can never take place, except in cases of sudden death, when the stomach is in full health, and the gastric secretion is surrounded by a dead organ. Dr. Mason Good,† after having stated the opinion of Mr. Hunter, observes, " It is true the stomach has been found thus eroded in one or two cases, where death has followed after long general illness; but, in such instances, it is probable that the stomach itself had been free from the general disease, or was not essentially affected by it."

The symptoms of the case which fell under my observation, and those which have been detailed by the German physician, indicate very clearly the existence of gastric disease. It is, I know, an admitted fact, that the dissolvent power of the gastric juice is in an inverse ratio to the muscular force of the parietes of the stomach. Those animals in which the parietes of that viscus are very thin, and almost entirely membranous, are those in which the gastric juice has the most force and activity. Whatever may be the power of the gastric juice, however, the *vitality* of the stomach enables it to resist its destructive action. It is the vitality of the tender and delicate worms termed *lumbrici*, which enables them to resist, for so long a time, during their residence in the stomach, the dissolvent power of the gastric fluid.

M. Andral has published many highly interesting observations upon the pathological anatomy of the digestive canal, and has thrown out many remarks which bear directly upon this subject. The original paper I have not had an opportunity of consulting. A translation of it is contained in the Medico-Chirurgical Journal, December, 1823.‡ Ulcerations follow the pimples and

* *Philosophical Transactions*, 1772, vol. 62, p. 447.
† *Study of Medicine*, vol. 1, p. 16.
‡ And in this Journal, July of the same year.—EDITORS.

pustules, with which the mucous membrane of the stomach and intestines is sometimes covered. It is suggested as a question by M. Andral, whether "the greatest analogy does not exist between the mode of the production of these ulcerations, and the development of certain ulcers of the mouth, which likewise owe their origin to small pustules known under the name of apthæ?" It frequently happens that the mucous membrane may for some time be the seat of inflammation, and that no solution of its continuity is detected. The occasional rapidity of its ulceration is probably connected with some idiosyncrasy, which is yet to be detected by pathological researches, and a careful consideration and comparison of the appearances discovered, with the symptoms exhibited during life, and the particular temperament and disposition of the patient. If living animals,* of the same age and strength, are poisoned by corrosive sublimate, we shall, at the end of forty-eight hours, discover only symptoms of inflammation of the mucous membrane in some, whilst in others ulceration will have taken place.

M. Chaussier† has sometimes detected ulceration of the mucous membrane of the stomach in lying-in women. The works of Bichat and Broussais contain much valuable and instructive matter upon the subject, and describe very accurately the appearances that are produced by disease of the stomach and contiguous parts, from the first blush of inflammatory action to positive destruction of texture.

In many points of view, the subject is worthy of investigation; and I have no doubt that, if it were customary to inspect the bodies of young children who perish with symptoms of gastric derangement, that we should detect the progress to positive destruction of texture in its different stages; and, by a due attention to the symptoms of each case, we might derive the gratifying power of employing our means of relief, before the disease with which we had to contend was beyond the reach of art. For another reason, also, the subject demands attention. The destruction of the stomach and contiguous parts, which is found in these cases, and which I have no doubt is the result of inflammation, perhaps neglected or overlooked in its first stages, is so very similar to the appearances that would be produced by acrid poisons, that, under particular circumstances, which may easily be conceived possible, unfounded suspicions might be entertained as to the real cause of the death of the child, by those who are ignorant of the fact of their occasional occurrence from disease.‡

*Seymour-place, Bryanstone-square.*

* M. Andral, *loco citato.*

† *Dict. des Sc. Med.* tome 32, p. 368, and tome 50, art. *Perforation.*

‡ We likewise beg leave to refer our readers to the work of M. Cruvelhier, reviewed by us in vol. xlviii. of this Journal.—Editors.

